# Pharmacokinetics of Amoxicillin in the Cat

**DOI:** 10.1111/jvp.70003

**Published:** 2025-06-02

**Authors:** Ilse R. Dubbelboer, Lena Olsén, Lena Pelander, Marlene Z. Lacroix, Lucie Claustre, Beatrice Roques, Carl Ekstrand

**Affiliations:** ^1^ Department of Pharmaceutical Biosciences Uppsala University Uppsala Sweden; ^2^ Department of Clinical Sciences Swedish University of Agricultural Sciences Uppsala Sweden; ^3^ INTHERES INRAE, ENVT, Université de Toulouse Toulouse France; ^4^ Department of Animal Biosciences Swedish University of Agricultural Sciences Uppsala Sweden

**Keywords:** antibiotic, disposition, feline, penicillin, plasma concentration

## Abstract

The pharmacokinetics and plasma protein binding of amoxicillin in cats has not been thoroughly investigated. In a single‐group sequential designed experimental study, amoxicillin was administered to six healthy cats intravenously, orally, and subcutaneously. Repeated blood samples were drawn after each administration, and amoxicillin concentrations were determined using High Performance Liquid Chromatography coupled to Triple Quadrupole Mass Spectrometry. Plasma amoxicillin data were subjected to population pharmacokinetic analysis, and pharmacokinetic parameters were estimated. The population clearance was 0.18 L/h∙kg, the volume of the central compartment was 0.12 L/kg, the highly perfused compartment was 0.009 L/kg, and the poorly perfused compartment was 0.002 L/kg. The bioavailability was 33% and 69% after oral and subcutaneous administration, respectively. After subcutaneous administration of a slow‐release formulation, there was absorption rate‐limited pharmacokinetics. The plasma protein binding was 0%–24%. The results increase the understanding of the amoxicillin pharmacokinetics in cats. Further studies combining the results with pharmacodynamic data and *in silico* simulations are warranted.

## 1 Introduction

Amoxicillin is a semisynthetic aminopenicillin with an enhanced antimicrobial spectrum compared to penicillin. It has been used since the 1970s, and clinical studies were performed in cats as early as then (Francis et al. 1978). Amoxicillin is considered to have a wide safety margin with a low risk of adverse effects (Isiordia‐Espinoza et al. 2015; Stegemann et al. 2007). Over time, the clinical efficacy of amoxicillin has been confirmed for several conditions in cats, for example, pyoderma, gingivitis, respiratory infections, bacterial cystitis, wound infections, and abscesses (Litster et al. 2007, 2012; Sturgess et al. 2001; Bywater et al. 1985; Senior et al. 1985; Stegemann et al. 2007; Wildermuth et al. 2012; Roy et al. 2007; Jones et al. 1994; Francis et al. 1978). Although those studies used different formulations, routes of administration, and some used amoxicillin in combination with clavulanic acid, the relatively large number of clinical studies suggests that amoxicillin is used commonly in cats.

Despite the common usage of amoxicillin, published pharmacokinetic data in cats are sparse. In 2007, Chicoine et al. published plasma concentration‐time data after the administration of an amoxicillin paste *per os* (Chicoine et al. 2007). Similar data were published more than 20 years ago after oral administration of amoxicillin and clavulanic acid, and more recently a pharmacokinetic study on amoxicillin and clavulanic acid administered by oral and intravenous routes was published (Vree et al. 2002; Yang et al. 2019). However, the pharmacokinetic profile of amoxicillin in cats without co‐administration with clavulanic acid deserves further attention. In addition, there are slow‐release formulations with amoxicillin available, and concentration‐time data of those appear to be missing for cats in the scientific literature. Moreover, the plasma protein binding of amoxicillin was not reported in the aforementioned studies. Investigating the fraction of the drug bound to plasma protein is essential to allow simulations of the antibacterial response to different dosing regimens. The aims of this study were (i) to derive plasma concentration‐time data after intravenous (IV), oral (PO) and subcutaneous (SC) administration, (ii) to apply a pharmacokinetic model to the data, (iii) increase the understanding of amoxicillin absorption and disposition in cats, and (iv) to determine the fraction of amoxicillin bound to plasma proteins in cats.

## 2 Materials and Methods

### 2.1 Animals

Six neutered, domestic, university‐owned cats (two males and four females) were included in the study. The cats were all 2 years old, and the median (range) bodyweight was 4.30 kg (3.96–5.70 kg). All cats were considered healthy based on full clinical examinations and on biochemical and haematological analyses. The cats were brought to the research facility at the National Veterinary School of Toulouse more than 1 year before the onset of the study. During drug administration and sampling, cats were held in a separate room and housed in individual stainless‐steel cages (0.7 ∙ 0.7 m). During the wash‐out, cats were group‐housed in a 23 m^2^ room. The room was enriched with scratching posts, cat trees, elevated platforms, beds with mats, a tower for the kibbles, brushes, balls, and ropes to play with and pheromones. The cats were fed commercial dry food (Vet Care Nutrition Cat Neutered Satiety Balance, Royal Canin, France). During pharmacokinetic assessment periods, the cats were fed 30 g feed 30–120 min before drug administration, 20 g before the first afternoon sampling, and 20 g before the last afternoon sampling. Water was available without limitation. For haematology and clinical chemistry, blood was collected in K3EDTA‐coated and lithium heparin‐coated tubes, respectively, and analysed at *Laboratoire Central de Biologie Médicale* at the National Veterinary School of Toulouse. The measured variables were: white blood cell count (WBC), red blood cell count (RBC), hemoglobin (HGB), mean cell volume (MCV), mean cell hemoglobin (MCH), platelets (PLT), microhematocrit (μHCT), and white blood cell differentials (neutrophils, band cells, lymphocytes, monocytes, eosinophils, basophils), glucose, urea, creatinine, total protein, albumin, aspartate aminotransferase (AST), alanine transaminase (ALT), and alkaline phosphatase (ALP). Any cat suspected to suffer from disease based on clinical examination (e.g., hyperthermia, enlarged lymph nodes, cardiac or respiratory anomaly, digestive problem, difficult to handle) or on bloodwork with focus on impaired hepatic or renal function was excluded from the study. In the event of clinical signs of allergy to amoxicillin (redness of the skin, swelling, dyspnoea) during the study, the cat was excluded.

### 2.2 Experimental Protocol

The study was designed as a single‐group experimental study with a sequential design including three different routes of administration of amoxicillin: IV, PO, and SC. The administrations were given in the same order for all cats: first PO, then SC, and last IV. The wash‐out period was 2 weeks between PO and SC administration and 4 weeks between SC and IV administrations. For IV administration, sodium amoxicillin (Amoxicilline Panpharma, Panpharma, France, diluted to 50 mg/mL) was administered via a catheter placed in the cephalic vein aiming at a dose of 10 mg/kg amoxicillin base (dose range 9.48–10.27 mg/kg). For PO administration, amoxicillin trihydrate tablets (50 mg amoxicillin or 57.5 mg amoxicillin trihydrate, Amoxibactin 50 mg, Dechra veterinary products, Great Britain) were administered followed by 2 mL water to assure delivery to the stomach. For SC administration, amoxicillin was administered at a dose of 15 mg/kg (17.2 mg/kg amoxicillin trihydrate; Trymox LA 150 mg/mL, Huvepharma, Bulgaria) was given. The experimental protocol was declared to the ethical Committee “Toxcomethique” (*Comité d'Ethique de Pharmacologie‐Toxicologie de Toulouse Midi‐Pyrénées—n°86*) and to the French Ministry of Research. It was authorized by the French Ministry of Research under the number #16789_2018091914238153 with five antibiotic administrations of amoxicillin or amoxicillin/clavulanate to eight cats. The diminution of cat number (6 instead of 8) and administration number by cats (3 administrations instead of 5) was declared to, and approved by, the animal welfare structure of INTHERES.

### 2.3 Blood Sampling Protocol

1 mL blood samples were drawn from one of the jugular veins (or a cephalic vein if the cat did not allow sampling from the jugular vein) and transferred into 1 mL tubes containing lithium heparin. Tubes were centrifuged at +4°C for 10 min at 3000 *g* before plasma was removed and stored at −20°C (less than 3 months) until amoxicillin plasma analyses.

For determination of amoxicillin plasma concentrations, blood was collected before administration, 5, 10, 40 min, 1.5, 3, 5, 7, 9, and 11 h after IV administration, before administration, 15, 30 min, 1, 2, 3, 5.5, 7, 9, 12, and 24 h after PO administration, and before administration, 15, 30 min, 1, 2, 3, 5.5, 7, 10, 24, 34, and 48 h after SC administration.

In order to prevent hematomas, a pentosan sulfate polyester (Hémoclar 0.5% Sanofi, France) was applied on the skin after blood sampling.

### 2.4 Analytical Method

Amoxicillin plasma concentrations were determined by an Ultra‐High Performance Liquid Chromatography system (Nexera LC40, Shimadzu, Japan) coupled to an 8045 triple quadrupole mass spectrometer (Shimadzu, Japan). Plasma samples (50 μL) were spiked with 400 μL of MeOH and 10 μL of IS amoxicillin‐d4 at 1 μg/mL in H_2_O and centrifuged for 10 min at 20,000 *g* and +4°C. The supernatant (350 μL) was evaporated under nitrogen at +45°C and reconstituted in 100 μL of H_2_O. The analytes (10 μL) were injected on a C18 column (Acquity HSS T3, 2.1 × 100 mm, 1.7 μm, Waters, Milford, MA, USA) with an H_2_O, 0.1% HCOOH/Acetonitrile (AcN) gradient elution (*t*0): 0% AcN; *t*(2 min): 60% AcN; *t*(2.5 min): 0% AcN; *t*(4 min):0% AcN. Amoxicillin and amoxicillin‐d4 were ionised by electrospray in positive mode (ESI+) and detected by multiple reaction monitoring mode (MRM) with the following transitions: amoxicillin *m*/*z* 366 > 114 (collision energy [CE] −20 V) and amoxicillin d4 *m*/*z* 370 > 114 (CE −25 V). The method was validated with a calibration curve ranging from 0.01 to 10 μg/mL and three quality control (QC) samples (0.025, 0.75 and 7.5 μg/mL). The LLOQ was defined as the lowest point on the calibration curve with a signal‐to‐noise ratio of at least 10:1. Intra‐day and inter‐day precision were evaluated using ANOVA and expressed as the coefficient of variation (CV%), calculated as the standard deviation of the measured concentrations divided by the mean. Recovery was assessed by calculating the mean measured concentration at the LLOQ (or QC level) and dividing it by the corresponding theoretical concentration. Plasma samples with concentrations exceeding 10 μg/mL were re‐assayed after a 1:10 dilution in blank plasma. This dilution step was validated using a QC sample at 50 ng/mL, which was also diluted 10‐fold in blank plasma. Amoxicillin was quantified using a linear model weighted by 1/*X* ^2^. The lower limit of quantification (LLOQ) was 0.01 μg/mL with a recovery of 99% and an inter‐day variability of 2%. The recoveries of QC ranged from 101% to 111% for the three levels of QC concentrations, and the mean intra‐day and inter‐day variability were less than 15%. Recovery for the 1:10 dilution was 120%, with an intra‐day precision of 5%.

### 2.5 Determination of Protein Binding

Amoxicillin plasma protein binding was determined with 100 μL plasma using Thermo Scientific Rapid Equilibrium Dialysis (RED) inserts and plates (Fisher Scientific, Illkirch, France). To determine amoxicillin unbound fraction (f_u_), one sample per cat and per route of administration as close as possible to the maximal plasma concentration was used. Briefly, 100 μL of cat plasma sample was added to the sample chamber, and 350 μL of dialysis buffer (PBS, pH 7.4) was added to the buffer chamber. The plate was covered with sealing tape and incubated at 37°C on an orbital shaker at approximately 250 rpm for 4 h. Amoxicillin concentration was determined in each chamber by LC/MS/MS as described previously. To prepare the samples for analysis, 50 μL of plasma (or buffer) was added to 50 μL of blank buffer (or blank plasma) and 300 μL of IS diluted in AcN to precipitate proteins. The amoxicillin/IS peak area ratio was used to determine the amoxicillin concentration, and f_u_% was calculated as the percentage of amoxicillin concentration in the buffer chamber divided by the amoxicillin concentration in the plasma chamber. When the calculated f_u_% exceeded 100% (observed in only two cats following oral administration, likely due to assay variability), protein binding (%B) was reported as 0% to avoid reporting implausible negative values.

### 2.6 Pharmacokinetic Analysis

For initial data exploration, to describe the individual pharmacokinetic (PK)‐profiles and to provide input to the population PK analysis, data was subjected to non‐compartmental analyses using PKanalix 2023R1 (Lixoft SAS, A Simulations Plus company). The analyses were performed separately for each administrative route using a linear up and log down integral method. A minimum of three observations were used for estimation of the slope of the terminal phase (elimination rate constant [*k*_*e*_]). The parameters Clearance (*Cl*), Volume of distribution (*V*_*d*_), *k*_*e*_, and area under the curve extrapolated to infinity (AUC_0‐ꚙ_) were estimated by the model. The parameters maximal observed concentration (*C*_max_) and time for the maximum observed concentration (*T*_max_) were observed. For population PK analysis, a non‐linear mixed effects (NLME) model was fitted to experimental data using Monolix 2023R1 (Lixoft SAS, A Simulations Plus company). One‐, two‐, and three‐compartment models with first‐order elimination were fitted to amoxicillin IV data. Model evaluation was performed by means of graphical inspection of diagnostic plots (individual fits, observed data vs predicted data, weighted residuals vs time, weighted residuals vs concentration and the visual predictive check [VPC]), parameter precision, and objective function values (OFVs), that is, minus twice the log of the likelihood (−2LL) and Bayesian Information Criteria (BIC). Observations below the lower limit of quantification (LLOQ) were censored, that is, any concentration between 0 and LLOQ was plausible. The final PK model was parameterized using Clearance (*Cl*), the volume of the central compartment (*V*_1_), the highly perfused compartment (*V*_2_), the poorly perfused compartment (*V*_3_), inter‐compartmental clearance from compartment *V*_1_ to compartment *V*_2_ (*Q*_1_) and inter‐compartmental clearance from compartment *V*_1_ to compartment *V*_3_ (*Q*_2_). A multiplicative (proportional) residual error model was used. The model was first fitted to IV data in order to correctly estimate *Cl, V*_1_, *V*_2_, *V*_3_, *Q*_1_, and *Q*_2_. Those parameters were then fixed, and the model was fitted to PO data and to SC data in order to estimate the lag time (*T*_*lag*_), absorption rate constant (*k*_*a*_), and the bioavailability (*F*). All parameters were assumed to be lognormally distributed except *F*, which was assumed to be logitnormally distributed.

The statistical model for the random effects was described by:

(1) $$\theta_{i}=\theta_{\mathrm{tv}}∙\exp\left(\eta_{i}\right)$$

where $\theta_{i}$ is the value of the pharmacokinetic parameters in the *i*th cat, $\theta_{\mathrm{tv}}$ is the typical population value of the parameter and η_i_ is the deviation from the corresponding population value associated to the *i*th cat. Random effects were excluded for *Q*_1_ and *Q*_2_ parameters due to the sparse nature of the date set and in order to increase parameter precision of remaining parameters. The standard deviation of the random effects (*ω*) reported by Monolix was then transformed to a coefficient of variation (CV%) using Equation (2):

(2) $$\mathrm{CV}\%=\sqrt{\exp\left(\omega^{2}\right)-1}∙100$$

The CV% was then used to express the inter‐individual variability (IIV). Shrinkage of the random effects (eta) toward the means was described as:

(3) $$\text{shrinkage}=1-\frac{\mathrm{var}\left(\eta_{r}\right)}{\omega^{2}}$$

where var.(*η*_*r*_) is the variance of the random effects. When shrinkage for eta was > 30%, the random component was not considered robustly estimated and should not be used for individual parameter estimates. Covariates (sex [categorical] and bodyweight [continuous]) were added one by one to each of the parameters of the model and evaluated for model improvement. For inclusion in the final model, the covariate should decrease both IIV and OFVs. Moreover, parameter precision should increase, the correlation between the covariate and the parameter should be significant (tested by Pearson's test) and the covariate estimate should be statistically significant separated from zero (tested by Wald's test). A *p*‐value less than 0.05 was considered significant.

## 3 Results

**FIGURE 1:**
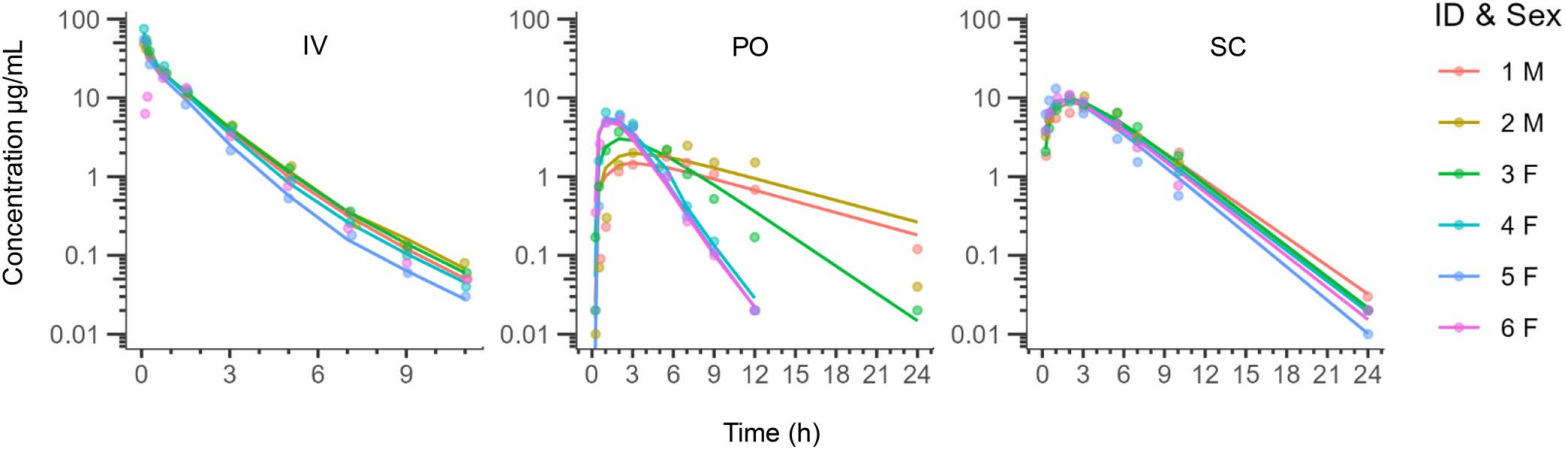
Semi logarithmic spaghetti plot showing observed (symbols) and model predicted (lines) plasma amoxicillin concentration‐time courses after 10 mg/kg intravenously (left plot, IV), 10 mg/kg orally (middle plot, PO) and 15 mg/kg subcutaneously (right plot, SC) to six cats. For one cat, the intravenous dose was injected perivascular so intravenous data from this cat was excluded.

No clinical adverse effects related to treatment were observed. Raw experimental data suggested perivascular administration instead of IV administration for one cat. The IV data from this cat were excluded from the PK analysis. Oral and SC data from that cat were included in the analyses. The plasma amoxicillin concentration‐time courses for all treatments are shown in Figure 1.

**FIGURE 2:**
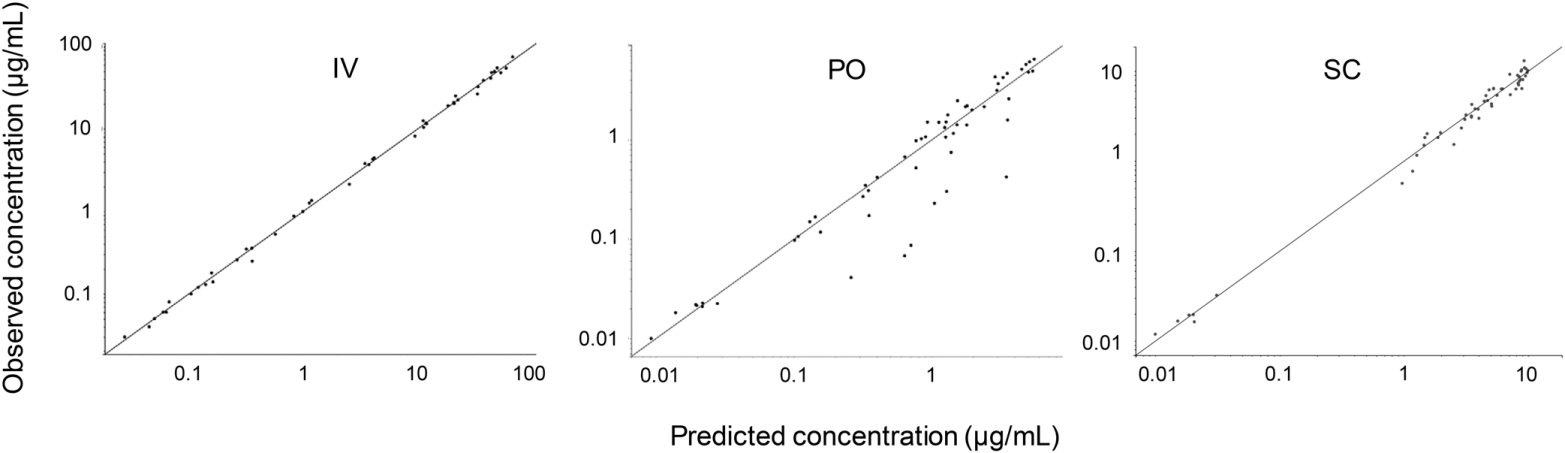
Godness of fit plots showing observed concentrations VS predicted concentrations after intravenous (IV) administration, oral (PO) administration and subcutaneous administration (SC) of amoxicillin to six cats. Filled black circles represent observed data. The solid line represent the line of unity (observation = prediction).

**FIGURE 3:**
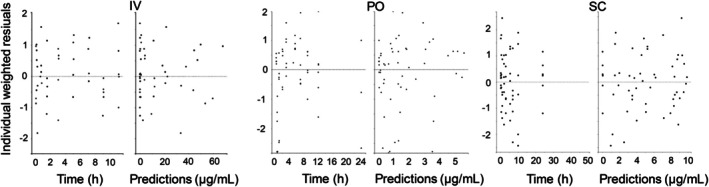
Goodness of fit plots showing Individual weighted residuals versus time and predicted concentration after intravenous (IV) administration, oral (PO) administration and subcutaneous administration (SC) of amoxicillin to six cats.

**FIGURE 4:**
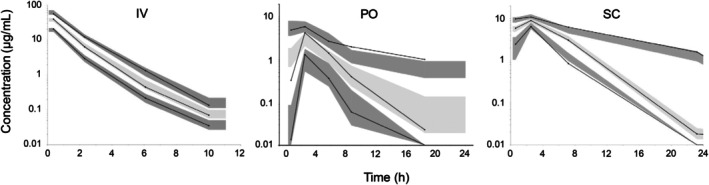
Visual predictive check after intravenous (IV) administration, oral (PO) administration and subcutaneous administration (SC) of amoxicillin to six cats. The solid lines represents the 10th, 50th, and 90th empirical percentile, respectively. The dark grey shaded areas are the 10th and the 90th prediction interval and the light grey shaded area is the median prediction interval.

The final population model was a three‐compartment model. The use of a three‐compartment model fitted to IV data instead of a two‐compartment model decreased −2LL by 57 units and BIC by 74 units. Visual inspection of diagnostic plots indicated a good fit of the model to experimental data. In a second step, the model was also fitted to experimental PO and SC data. Shrinkage were less than 30% for the parameters *Cl*, *V*_1_, *F*_po_, *T*_lagpo_, *k*_apo_, *F*_SC_, *T*_lagSC_, and *k*_asc_. For the parameters *V*_2_ and *V*_3_, shrinkage was > 30%. Pharmacokinetic parameters from the non‐compartment analysis are shown in Table 1 and model parameters from the population compartment analysis are shown in Table 2. During model building, sex was determined to be a viable covariate for *k*_*a*_ following PO (*k*_apo_) administration. Inclusion of sex as a covariate decreased the −2LL by 9 units and BIC by 7 units. The relative standard error for the *k*_apo_ parameter decreased from 36.5% to 22.3% and the IIV decreased from 108.6% to 40.9%. Without the use of the covariate “sex” the population value for *k*_apo_ was 0.34 1/h. There was a correlation between the covariate and the parameter (*p* = 0.01) and the covariate parameter was statistically separated from zero (*p* < 0.001). Model fit is shown in Figure 1. Diagnostic plots are shown in Figures 2, 3, 4.

**TABLE 1:** Amoxicillin pharmacokinetic parameters estimated by non‐compartmental analysis after intravenous, oral and subcutaneous administration of amoxicillin to six cats.

Parmeter	Unit	Cat 1	Cat 2	Cat 3	Cat 4	Cat 5	Cat 6	Median
*Cl*	L/kg·h	0.18	0.17	0.17	0.16	0.23	—	0.17
*V*_*d*_	L/kg	0.36	0.55	0.28	0.33	0.47	—	0.36
*C*_maxPO_	mg/L	1.8	2.5	4.3	6.5	5.8	5.2	4.4
*C*_max SC_	mg/L	7.4	10.4	10.2	8.9	13.1	10.9	10.3
*T*_maxPO_	h	5.5	7.1	1.1	3.3	2.1	2.1	2.5
*T*_maxSC_	h	3	3	2	2	1	2	2
AUC_0‐ꚙIV_	mg∙h/L	55.7	57.7	62.0	65.3	43.7	—	57.4
AUC_0‐ꚙPO_	mg∙h/L	17.5	24.7	21.4	21.6	18.8	17.2	20.1
AUC_0‐ꚙSC_	mg∙h/L	51.1	63.0	62.0	54.8	48.8	55.0	54.9
*k*_*eIV*_	1/h	0.50	0.31	0.58	0.47	0.49	—	0.49
*k*_*ePO*_	1/h	0.15	0.26	0.21	0.59	0.52	0.52	0.39
*k*_*eSC*_	1/h	0.27	0.31	0.33	0.30	0.28	0.29	0.29
*t*_*1/2zIV*_	h	1.4	2.3	1.2	1.5	1.4	—	1.4
*t*_*1/2zPO*_	h	4.7	2.7	3.3	1.2	1.3	1.3	2.0
*t*_*1/2zSC*_	h	2.6	2.3	2.1	2.3	2.6	2.4	2.4

*Note: Cl* is the clearance, *V*_*d*_ is the volume of distribution, *C*_maxPO_ and *C*_max SC_ are the highest observed plasma concentration after oral and subcutaneous administration, *T*_maxPO_ and *T*_maxSC_ is the time for *C*_maxPO_ and *C*_max SC_, AUC_0‐ꚙIV_, AUC_0‐ꚙPO_ and AUC_0‐ꚙSC_ are the area under the plasma concentration‐time courve from the time 0 h to infinity after intravenous, oral and subcutaneous administration, *k*_eIV_, *k*_ePO_ and *k*_eSC_ are the elimination rates after intravenous, oral and subcutaneous administration and *t*_*1/2zIV*_, *t*_*1/2zPO*_ and *t*_*1/2zSC*_ are the terminal half lives after intravenous, oral and subcutaneous administration.

**TABLE 2:** Amoxicillin pharmacokinetic model parameters and their precision estimated by non‐linear mixed effect modelling (compartmental analysis) after intravenous, oral, and subcutaneous administration of amoxicillin to six cats.

Parameter	Unit	Typical value	R.S.E. (%)	I.I.V. (%)
*V*_*1*_	L/kg	0.12	12.7	13.31
*V*_*2*_	L/kg	0.092	0.0003	0.0001
*V*_*3*_	L/kg	0.021	0.0002	0.00004
*Cl*	L/kg·h	0.18	4.97	8.03
*Q*_*1*_	L/kg·h	0.39	15.8	—
*Q*_*2*_	L/kg·h	0.009	7.47	
Residual error parameter		0.11	12.2	—
*T*_lagpo_	h	0.25	3.9	108.6
*T*_lagsc_	h	0.06	81.4	106.3
*k*_apo_	1/h	0.6	20.5	40.9
*K*_apo_covariate sex “male*”*_		−1.7	22.3	—
*K*_asc_	1/h	0.31	2.8	5.7
*F*_oral_	%	33	8.2	12.8
*F*_sc_	%	69	8.5	2.3

*Note: V*_1_, *V*_2_, *V*_3_, are the volumes of the central, the highly perfused and the scarcely perfused compartments, *Cl* is the total body clearance, *Q*_1_, *Q*_2_ are the inter‐compartmental distribution clearance between *V*_1_ and *V*_2_ and *V*_1_ and *V*_3_. *T*_lagpo_, *T*_lagsc_, are the lag‐time after oral administration and the lag‐time after subcutaneous administration. *k*_apo_, *K*_asc_, are the absorption rate constant after oral administration and the absorption rate constant after subcutaneous administration. *F*_oral_ and *F*_sc_ are, the oral bioavailability and the subcutaneous bioavailabilty. *K*_apo_covariate sex “male”_ is the parameter describing the difference in parameter value dependent on the covariate “sex”, R.S.E is the relative standard error of the typical value and IIV (%) is the inter‐individual variability defined as the coefficient of variation (CV%) of the random effects.

### 3.1 Protein Binding

The median (range) fraction amoxicillin protein bound was 10.5% (0%–24%). Divided into routes of administration, the median (range) fraction protein bound was 11% (8%–13%), 1.5% (0%–17%) and 11.5% (6%–24%) for the IV, PO, and SC routes, respectively (Table 3).

**TABLE 3:** Protein binding data and corresponding total plasma concentrations following administration of amoxicillin to six cats.

Route of administration	Cat Id	Protocol sampling time (h)	Amoxicillin total plasma concentration (μg/mL)	%Fu	%B
Oral	1	7	1.50	100%	0%^a^
	2	5.5	2.16	97%	3%
	3	1	2.15	100%	0%
	4	2	6.10	86%	14%
	5	1	4.95	83%	17%
	6	1	4.80	100%	0%^a^
Median %					1.5%
Subcutaneous	1	2	6.42	85%	15%
	2	3	10.4	76%	24%
	3	2	10.19	89%	11%
	4	2	8.87	94%	6%
	5	2	10.68	88%	12%
	6	2	10.94	91%	9%
Median %					11.5%
Intravenous	1	0.17	32.66	88%	12%
	2	0.17	41.55	92%	8%
	3	0.17	39.03	88%	12%
	4	0.17	49.87	90%	10%
	5	0.67	19.19	87%	13%
	6	0.17	10.32	90%	10%
Median %					11%

^a^ Implausible negative value %Bound set to 0%.

## 4 Discussion

This is the first scientific report on the pharmacokinetics of amoxicillin after IV, PO, and SC administration, and protein binding in cats. It is also the first scientific report on the amoxicillin plasma concentration‐time course after SC administration of a slow‐release formulation to cats. After SC administration, all cats displayed absorption rate limited elimination (flip‐flop kinetics) as determined by increased time to maximum observed concentration and longer half‐life. This was expected because this product was designed to reduce absorption rate and extend the duration of the plasma concentration‐time curve.

After IV administration of amoxicillin, the plasma concentrations in the present study were consistently higher than in a similar study by Yang et al. (2019), leading to a roughly 2‐fold higher IV AUC_IV_ (57.6 mg∙h/L vs. 23.87 mg∙h/L). The half‐life was comparable between studies. Consequently, the calculated *Cl* (present: 0.17 L/kg∙h, Yang: 0.453 L/kg∙h) and *V*_*d*_ (present: 0.36 L/kg, Yang: 0.56 L/kg) parameters after IV administration were lower in the present study. The main differences between the studies are, besides no co‐administration of clavulanic acid, the use of neutered and slightly heavier cats in the present study. Moreover, although Yang et al. (2019) sampled the cats more frequently, the first sample was drawn 5 min post administration in both studies. In general, dose, mode of administration, sampling protocol, analytical method, and performance or drug formulation might influence the AUC (Hoffman et al. 2001), thus explaining the differences between studies. In other species, plasma amoxicillin concentration‐time courses are similar to the present study, and *Cl* was estimated to be 0.2, 0.37, and 0.27 L/kg·h in dogs, pigs, and horses, respectively (Küng and Wanner 1994; Agersø and Friis 1998a; Ensink et al. 1992; Montesissa et al. 1988). Moreover, the *t*_*1/2z*_ was similar between the current study and the study by Yang et al. (2019), so the most probable explanation for the differences in *Cl* and *V*_*d*_ values is inter‐study variability.

The bioavailability (F) after PO administration was lower in this study (33%) than previously reported 77% in cats (Yang et al. 2019). Our cats were in the fed state, whilst the cats in the Yang study were fasted. In other species, bioavailability varies from 20% to 30% in pigs, 67% in horses, to 64% to 77% in dogs (Decundo et al. 2024a; Küng and Wanner 1994; Montesissa et al. 1988), and feeding has been shown to have an effect on amoxicillin bioavailability in dogs, pigs, and humans (Watson et al. 1986; Agersø and Friis 1998a; Decundo et al. 2024b; Eshelman and Spyker 1978; Welling et al. 1977). It is thus likely that the difference in bioavailability was due to the fed state of the cats in the present study.

Flip‐flop kinetics after PO administration were observed in two male cats in this study, which required the use of sex as a covariate on the *k*_*a*_ parameter in the compartmental modeling. Flip‐flop kinetics for amoxicillin after PO dosing have also been observed in other species, such as humans, pigs, and goats (Welling et al. 1977; Agersø and Friis 1998a; Paintaud et al. 1992; Carceles et al. 1995), and it is well known that amoxicillin can display non‐linear absorption in humans. Several suggestions for these observations have been made. As both AUC_PO_ and bioavailability were similar for all cats (including those displaying flip‐flop kinetics) after PO dosing, we disregarded solubility issues associated with higher absolute amoxicillin dose or degradation in the stomach due to low feline gastric pH (~2–2.5) (Thambavita et al. 2017; Hofsäss and Dressman 2020; Telles et al. 2022). Most likely, the combination of the fed state with high inter‐individual variability in gastric emptying time led to the variability in PO half‐life. This is supported by the lack of flip‐flop kinetics of amoxicillin in the fasted cats by Yang et al. (2019) and the decreasing oral absorption rate of amoxicillin in humans in the fed state (Eshelman and Spyker 1978). The large inter‐individual variability in feline gastric emptying time was observed with non‐digestible pH capsules (Telles et al. 2022) and polyethylene spheres of different sizes (Sparkes et al. 1997; Chandler et al. 1997, 1999), and food in the stomach prolongs the gastric emptying time (Chandler et al. 1997). Gastric emptying rate was not sex‐related in cats (Goggin et al. 1998), making it unlikely that the flip‐flop kinetics are due to the sex of the cat. Furthermore, despite similar doses per kg body weight, the cats displaying flip‐flop kinetics after PO dosing did receive a higher absolute dose (1.25 tablet) than the other 4 cats (1 tablet). Consequently, certain conclusions are difficult to draw based on the small study population. Sex as a covariate for the *k*_*a*_ parameter might be a proxy for the above‐discussed mechanisms (gastric emptying, absolute dose dissolution) which cannot be captured with empirical modeling. It is likely that this covariate would not be significant in a larger population of cats, why this covariate should be interpreted with caution.

As a higher variability in half‐life and a possible prolongation of half‐life after a PO dose were observed with food than without food, these effects should be studied during a complete treatment period, with multiple doses, to assess the impact on the effectivity of the drug. Additional studies comparing the AUC in a cross‐over study with both fed and fasted cats would provide more information about the effect of feeding on amoxicillin absorption. Physiologically based pharmacokinetic modeling can be used to elucidate the cause of the flip‐flop kinetics and evaluate the effect of food, solubility, and degradation on amoxicillin PK in cats following oral administration.

Protein binding is generally assessed by spiking blank plasma across a range of concentrations in pooled or individual plasma. However, this method requires additional blood sampling from each cat or the use of blank plasma from other cats. Since collecting blood samples from cats is challenging, the second aliquot from the pharmacokinetic experiment was used to minimize blood sampling for each cat. The use of frozen plasma has been shown to produce different values for the dissociation constant and the maximum binding capacity of flunixin meglumine in pigs (Buur et al. 2009). In contrast, freezing has not been shown to affect the free fraction of imipramine, lidocaine, diazepam, and phenytoin in human plasma (Morse et al. 1985). Using the method in the current study with frozen plasma, the median degree of amoxicillin bound to plasma proteins was 10.5% (range 0%–24%) which is consistent with what has been reported in dogs (0%–24%) and pigs (24%), but lower than in horses (37%–38%) (Agersø and Friis 1998b; Vegas Cómitre et al. 2021; Montesissa et al. 1988). This comparison does not consider potential non‐linearity in protein binding nor do differences due to methods to determine the degree drug bound to plasma proteins. The similar degree of protein binding between species does, however, suggest an accuracy in the results from the current study.

The main limitation of this study was the low number of study objects (two male and four female cats) at the same age (2 years old) which limited the range of variability in the studied population and decreased the interpretation to a clinical population. It also provided fewer observations than a larger study population would have done, and therefore the precision in pharmacokinetic parameters was more limited. The sampling protocol also had limitations. There was a relatively wide range of time between feeding and treatment. Reducing this interval for standardization in future studies would address one potential limitation of the current study. In addition, an earlier sample than after 5 min would possibly increase the chances of characterizing the initial distribution more precisely, leading to a more adequate back extrapolation and estimation of the plasma concentration at time = zero. More frequent sampling during the experimental legs would also have generated a richer dataset allowing more precise parameter estimation. However, considering the blood volume of a cat, the amount of blood withdrawn had to be restricted, which made more frequent sampling impossible. Finally, the study was performed on healthy cats, and it is known that disease, for example, inflammation, might influence the pharmacokinetic profile in a patient (Stanke‐Labesque et al. 2020).

This study has shown that amoxicillin concentrations decrease rapidly in cats after IV and PO administration and the fraction protein bound drug was low. Amoxicillin is a time‐dependent antibiotic and maintaining the free concentration above MIC at the target site over time is essential for therapeutic effects (de Velde et al. 2018). The antibacterial effect varies with the extent of time the concentration is above MIC in a dosing interval but around 50% has been a suggested point estimate for bactericidal effects using penicillin (Drusano 2004). At lower exposure, 30%–40% of the time above MIC, the effect is bacteriostatic. The short plasma half‐life suggests that amoxicillin possibly could be administered more than twice daily to maintain antibacterial activity during the entire dosing interval, depending on the sensitivity of the bacteria. Using SC administration of slow release formulations could possibly reduce the dose rate to once daily. More specific dose recommendations could be provided if the data from this study was combined with pharmacodynamic data in order to do computer simulations of treatment efficacy.

## Author Contributions

Carl Ekstrand, Lena Pelander, Lena Olsén, and Beatrice Roques planned the study. Beatrice Roques, Marlene Z. Lacroix, and Lucie Claustre performed the experiment and analyzed plasma samples. Ilse R. Dubbelboer, Carl Ekstrand, Lena Pelander, and Lena Olsén analyzed the data. Ilse R. Dubbelboer and Carl Ekstrand drafted the manuscript. All authors revised the manuscript and approved the final version of the manuscript.

## Ethics Statement

The experimental protocol was declared to the Ethical Committee “Toxcomethique” (*Comité d'Ethique de Pharmacologie‐Toxicologie de Toulouse Midi‐Pyrénées—n°86*) and to the French Ministry of Research. It was authorized by the French Ministry of Research under the number #16789_2018091914238153 with five antibiotic administrations of amoxicillin or amoxicillin/clavulanate to eight cats. The diminution of cat number (6 instead of 8) and administration number by cats (3 administrations instead of 5) was declared to, and approved by, the animal welfare structure of INTHERES.

## Conflicts of Interest

The authors declare no conflicts of interest.

## Data Availability

The data that support the findings of this study are openly available in the Swedish National Data Service at https://doi.org/10.5878/905y‐hq69.
